# Results of Simultaneous Unicompartmental Knee Arthroplasty and Anterior Cruciate Ligament Reconstruction: A Systematic Review

**DOI:** 10.3390/jcm10194290

**Published:** 2021-09-22

**Authors:** Erika Albo, Stefano Campi, Biagio Zampogna, Guglielmo Torre, Giuseppe Francesco Papalia, Lorenzo Alirio Diaz Balzani, Anna Maria Alifano, Rocco Papalia, Vincenzo Denaro

**Affiliations:** Department of Orthopaedic and Trauma Surgery, Campus Bio-Medico University of Rome, 00128 Rome, Italy; e.albo@unicampus.it (E.A.); b.zampogna@unicampus.it (B.Z.); g.torre@unicampus.it (G.T.); g.papalia@unicampus.it (G.F.P.); l.diaz@unicampus.it (L.A.D.B.); a.alifano@unicampus.it (A.M.A.); r.papalia@unicampus.it (R.P.); denaro@unicampus.it (V.D.)

**Keywords:** ACL injury, medial knee osteoarthritis, unicompartmental knee arthroplasty, ACL reconstruction, simultaneous surgery, outcomes

## Abstract

This systematic review aimed to investigate the clinical and functional outcomes and complication rate of simultaneous anterior cruciate ligament reconstruction (ACLR) and unicompartmental knee arthroplasty (UKA). A systematic search in PubMed–Medline, Cochrane Library, and Google Scholar was carried out to identify eligible randomized clinical trials, observational studies, or case series that reported on clinical and functional results of combined ACLR and UKA in adults with a unicompartmental knee osteoarthritis and ACL deficiency. Four retrospective studies and three prospective studies were included in this review. A total of 169 patients were included with a mean follow-up of 6.3 years. The Mean Oxford Knee Score improved from 29.4 to 43.9 at the final follow-up. All the other reported scores significantly improved after surgery. The overall revision rate was 3.5%. The MINORS score ranged from 8 to 14. Association analysis of MINORS score and year of publication, through Pearson’s coefficient, showed no significant association (*p* = −0.089). Simultaneous ACLR and UKA is a safe procedure with a significant postoperative improvement of functional and clinical outcomes for patients with ACL injury that complain of knee instability and isolated medial compartment pain.

## 1. Introduction

Medial knee osteoarthritis (OA) is a common condition affecting the quality of life of patients and limiting the ability to perform sports activities [1]. In addition, from 33% to 70% of young active subjects with an untreated primary ACL injury complain of medial femorotibial pain [2]. In an ACL deficient knee, the recurrent posterior subluxation of the femur typically leads to joint degeneration and wear of the posteromedial cartilage of the tibial plateau, increasing tenfold the risk of OA compared to the uninjured population [3]. The best option for the treatment of unicondylar end-stage knee OA and ACL injury is still debated, especially in young patients. Different strategies have been proposed, including ACL reconstruction (ACLR), high tibial osteotomy (HTO) with or without ACLR, unicompartmental knee arthroplasty (UKA) with or without ACLR and total knee replacement (TKR) [4,5,6]. In a recent study, ACLR combined with HTO showed a threefold higher rate of complications compared to UKA, mainly related to failure of the graft [7]. UKA guarantees bone stock preservation, faster recovery and better long-term functional results compared to TKA [8]. Nevertheless, when performed in ACL-deficient knees, UKA has a higher failure rate due to the altered joint kinematics with a recurrent anterior translation of the tibia in relation to the femur [9]. According to some authors, the increased motion may cause a higher polyethylene wear and consequent osteolysis [10,11,12]. To overcome this issue, restoring knee stability with a combined UKA and ACLR is crucial. ACLR alone is already a validated procedure with complete recovery and return to high level sports activity [13,14]. A staged or simultaneous ACLR combined with medial compartment UKA has been performed more frequently in recent years. The procedure requires experience with both surgical techniques an adequate patient selection.

Given the possible advantages of this technique and the increased interest manifested in orthopedic practice, the purpose of this review is to evaluate the current evidence on simultaneous ACLR and UKA, focusing on clinical outcome and complications.

## 2. Materials and Methods

The present systematic review was performed in adherence to the Preferred Reporting Items for Systematic reviews and Meta-Analyses (PRISMA) guidelines, following a predefined protocol registered with PROSPERO (CRD42020182683) [15].

### 2.1. Eligibility Criteria

Randomized controlled trials (CRT), prospective cohort studies (PCS), retrospective case–control studies (RCS), and case series (CS) published in peer-reviewed journals were considered for inclusion. Case reports, biomechanical reports, technical notes, letters to editors, instructional courses, cadaver studies, animal or in vitro research, systematic reviews and meta-analyses were excluded from the review process. Papers were included whether they reported on clinical and radiological results of simultaneous ACLR and UKA in adults with an ACL injury and unicondylar knee osteoarthritis. Articles focused on staged surgical procedures were considered ineligible. Moreover, according to the language skills of the authors, articles in English, Italian and Spanish were investigated.

### 2.2. Search Methods for Identification of Studies

A comprehensive search was performed through PubMed–Medline, the Cochrane Library and Google Scholar databases, including papers from the inception of each database until June 2021. A combination of free-text terms and Medical Subject Headings (MeSH) were searched in titles and abstracts. For the development of the search strategy, Boolean logic operators were applied to the following keywords: (“anterior cruciate ligament” OR “anterior cruciate ligament injury” OR “anterior cruciate ligament tear”) AND (“unicompartmental knee osteoarthritis” OR “unicondylar knee osteoarthritis”) AND (“anterior cruciate ligament reconstruction” OR “anterior cruciate ligament surgery” OR “surgical procedures” OR “arthroscopy” OR “operative” OR “treatment” OR “management”) AND (“unicompartmental” OR “unicondylar” AND “knee prosthesis” OR “knee arthroplasty” OR “knee replacement”). After duplicate removal, two independent reviewers (E.A. and S.C.) screened the retrieved articles by titles and abstracts, excluding studies without abstract or meaningful information. An accurate full-text reading of relevant papers was performed to confirm their eligibility. The senior author (R.P.) intervened to reach a final decision if the reviewers disagreed about the inclusion of a study. The selected articles, their references and the ineligible studies were reviewed and discussed by all the authors to minimize the risk of bias. Moreover, further potentially eligible studies were manually retrieved among the reference lists of the included papers and the relevant systematic reviews.

### 2.3. Data Collection Process

A predetermined form was prepared for data extraction which was independently performed by two reviewers (E.A. and S.C.). For each article included, the following data were extracted and summarized in tables: authors, year, study design, level of evidence, demographic characteristics of the study groups, mean follow-up, clinical and functional outcomes, type of prosthesis, surgical technique and complications. The primary outcome measure of this study was the effectiveness of the simultaneous ACLR and UKA treatment in terms of clinical and functional outcome. The rate of complications at the final follow-up was the secondary outcome.

### 2.4. Methodological Quality Assessment

The level of evidence (LOE) of the included studies was assessed according to the Oxford criteria. The quality of each investigation was independently evaluated by two reviewers (E.A. and B.Z.) using the Methodological Index for Non-Randomized Studies (MINORS) score [16]. The tool allows us to appraise the following domains: clearly stated purpose, the inclusion of consecutive subjects, prospective data collection, endpoints appropriate to the purpose of the study, unbiased assessment of the study endpoints, follow-up period appropriate for the study, loss to follow-up of less than 5%, prospective calculation of the study size. For comparative studies, the MINORS score includes four different specific items: adequate control group, contemporary group, baseline group equivalence, and adequate statistical analysis. Each item was scored from 0 to 2 points, reaching a global score of 16 points for non-comparative studies and 24 points for comparative studies with an ideal research design and methodological quality. We also employed the Pearson’s test to evaluate the possible improvement of the quality of the investigations through the years.

## 3. Results

### 3.1. Search Results

**Figure 1:**
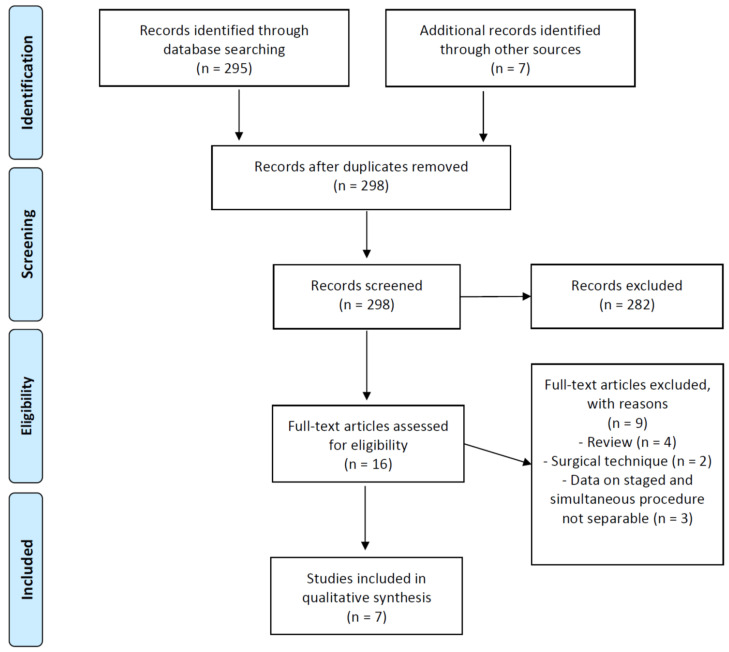
PRISMA flow-chart of included studies.

Starting from the 298 studies identified after database search and duplicate removal, 282 papers were excluded on the basis of titles and abstracts. After full-text reading of the potentially eligible papers, nine more studies did not fulfill the inclusion criteria: four were reviews of the literature, two reported on surgical technique without giving outcomes information and three showed not-separable data of populations that underwent staged or simultaneous ACLR and UKA. At the end of the selection process, seven papers were included in this systematic review (Figure 1) and data extraction was performed. In the paper of Kennedy et al. [17], a cohort of patients who underwent staged ACLR and UKA was included. We excluded data related to this subgroup, as they did not meet the inclusion criteria.

### 3.2. Study Characteristics and Demographic Details

Of the seven included studies, two were prospective [17,18] and four were retrospective [19,20,21,22] of level of evidence (LOE) IV. One was a retrospective cohort study with comparison group of LOE III [23].

The investigations were published between 2012 [19] and 2021 [22]. The overall number of combined procedures amounted to 169, with 169 patients involved. Within the included studies, the number of subjects varied from 8 [20] to 58 [17], with a mean age ranging from 44 [19] to 54 [21] years. The average follow-up was 6.3 years, ranging from 3.8 [22] to 14.6 [20] years. Further study characteristics are summarized in Table 1.

### 3.3. Preoperative Assessment

#### 3.3.1. Surgical Indications and Preoperative Symptoms

The majority of patients included were diagnosed with primary ACL lesion and concomitant medial compartment symptomatic osteoarthritis (OA). Tian et al. [18] also considered active patients with isolated medial OA and acute ACL injury that happened within 6 weeks during sports activity.

The primary complaints of all the patients were knee instability and pain referred at the medial compartment. Some authors highlighted that patients with a higher grade of OA (Ahlbäck’s type 3 and 4) complained of [15,19,20,22] pain as the most debilitating symptom, while instability was predominant in subjects with a lower degree of articular degeneration (Ahlbäck’s type 2) [21].

#### 3.3.2. Clinical and Radiological Examination

Manual laxity tests were performed to evaluate the ACL insufficiency. The diagnosis was verified by magnetic resonance image (MRI) in three studies [21,22,23], whereas other authors did not use it as a standard preoperative diagnostic tool [18,19,20].

Ventura et al. [21] quantified the anteroposterior laxity with an instrumented laxity test using the KT-1000 arthrometer (MEDmetric Corporation, San Diego, CA, USA). They showed significantly fewer anteroposterior translation from a preoperative mean side-to-side laxity of 5.7 mm to 2.8 mm after surgery (*p* < 0.001). Tian et al. [18], with a KT-2000 arthrometer, found a postoperative side-to-side difference inferior to 3 mm. Tinius et al. [19] made a similar evaluation demonstrating a reduction of maximal anterior translation at less than 5 mm in 89% of patients, using the Rolimeter (Aircast).

Standard radiographs and long-leg standing radiographs were sufficient to confirm the osteoarthritic pattern and evaluate lower limb alignment. Some authors used additional projection to verify the integrity of other articular compartments and the reducibility of the varus deformity. In particular, skyline views of the patella, Rosenberg view, and varus–valgus stress radiographic studies were used [18,19,20,23]. Kennedy et al. [17] did not perform a radiological analysis, indicating this as the main limitation of their investigation.

The majority of patients were diagnosed with bone-on-bone medial OA. Some authors used the Ahlbäck criteria to classify the degree of OA [19,20,21,22]. Among these, Iriberri et al. [20] and Ventura et al. [21] included, also, patients with grade 2 and 3 OA complaining of medial knee pain. In one paper, all patients had grade 4 OA according to the Kellgler–Lawrence classification [23].

As an additional evaluation, only Tecame et al. [23] classified the varus deformity through the “Thienpont and Parvizi” classification, including patients of Type IA–PMOA.

Moreover, Iriberri et al. [20] measured the anterior tibial translation (ATT) as an indirect sign of ACL deficiency. They verified that there was no significant reduction of ATT after surgery with a mean ATT value change from 1.6 mm (−5 to 8) to −0.9 mm (−10 to 8).

### 3.4. Surgical Technique

Preliminary knee arthroscopy was performed to confirm the diagnosis and the indication to the combined procedure in five studies [18,19,20,22,23].

In one study, the ACLR was carried out first and, subsequently, the arthroplasty was implanted [22]. Iriberri at al. [20] performed the bone tunnels for the ACLR after the UKA bone cuts and with the trial implants in position. In two studies, on the other hand, the bone tunnels were performed after the bone cuts [18,21]. Tecame et al. [23] performed the femoral tunnel before the bone cuts and then the tibial tunnel. The graft was passed through the tunnels after the positioning of the components [21,23], while in two studies the graft was pulled after component placement [18,20].

A trans-tibial technique for drilling the femur tunnel was reported in most papers [18,19,21,22]. One paper reported the out–in technique [20] and one study used the anteromedial portal for drilling the femoral tunnel [23].

The graft chosen for ACLR was mainly a four-stranded hamstring autograft except in one study [17], where both hamstrings and bone–patellar–bone autograft were used. The distal fixation of the graft was achieved through an interference screw in four studies [19,21,22,23]. In one study, a staple was used alone or with a screw for tibial fixation [20] and Tian et al. [18] used the Intrafix tibial fastener system (DePuy Mitek). Femoral graft fixation was more heterogeneous: EndoButton CL (Smith & Nephew, Memphis, Tenn) [18,23], TransFix^®^ pin (Arthrex Germany GmbH) [19], Rigid-Fix device (DePuy Mitek, Raynham, MA, USA) [21,22], staple or interference screw [20], Retrobutton (Arthrex, Naples, FL, USA) [21].

Cemented components were used in five studies [18,19,20,21,23], while both cemented and cementless fixation was performed in two series [17,22]. Data are summarized in Table 2.

### 3.5. Rehabilitation Protocols

Three authors described the main phases of the rehabilitation protocol [18,21,23] (Table 3). Partial weight-bearing with the assistance of crutches was allowed from the day after surgery, together with strengthening exercises and restoration of ROM. Tecame et al. [23] avoided knee flexion greater than 90° for the first week and used a brace without limitation of ROM during the initial phase of rehabilitation. Full weight-bearing started from the 2nd postoperative week in one study [18], while the other protocols introduced it one month after surgery. Proprioception exercises were allowed after four weeks.

### 3.6. Clinical and Radiological Outcomes

Internationally validated scores presented by more than one study were evaluated in the review process, in particular: Knee Society Score (KSS) in four studies [18,19,21,23], Oxford Knee Score (OKS) in four studies [17,18,21,22], Western Ontario and McMaster Score (WOMAC) in three studies [20,21,23], Tegner activity scale level (TAS) in two studies [17,18], Knee Osteoarthritis Outcomes Score (KOOS) in two studies [21,22]. EuroQol-visual analogue scales (EQ-VAS) [22] and Visual Analogue Scale (VAS) [20] for pain were only reported by one author, respectively. Furthermore, joint range of motion (ROM), the variation of anterior tibial translation (ATT), and extension and flexion deficit after the index surgery and at follow-up were evaluated in two investigations [18,19].

Radiological evaluation at follow-up was conducted on standard AP and lateral weight-bearing X-rays in order to evaluate tibial slope and any presence of radiolucencies or loosening of the components in almost all the studies. For the evaluation of lower limb alignment, a full-length weight-bearing radiograph was taken [18,19,22,23]. The mean KSS and the mean OKS improved from a mean value of 106 to 163 and 29 to 44, respectively.

#### Detailed Outcomes

Tian et al. [18] implanted a mobile-bearing prosthesis in a population of 28 patients who underwent combined ACLR. At a mean follow-up of 4.3 years, all patients perceived knee stability. TAS, KSS, and OKA improvements were statistically significant (*p* < 0.05). There was a positive correlation between a higher posterior slope of the tibial component and better ROM. The mean ROM in the sagittal plane was 123.5° ± 2.8° with a mean tibial slope value of 3.9° ± 1.2°. A similar tibial slope of 3.7° ± 1.6° was measured in a previous retrospective study with a cohort of 27 patients and the same significant correlation was confirmed with ROM [19]. At a mean 4.2-year follow-up, KSS increased from 77.1 ± 11.6 pre-injury to 166.03 ± 12 (*p* < 0.01). About KSS subcategories, knee score reached 83.2 ± 6.8 and function score 82.7 ± 8.2, similar to the other studies. There was not a statistically significant difference in outcome measures between males and females and between patients with or without radiolucent lines at the final radiological evaluation. The leg alignment changed from a preoperative varus deformity of 2.7° ± 1° to a valgus deformity of 3.9° ± 1°. After surgery, the authors demonstrated a reduction of maximal anterior translation at less than 5 mm in 89% of patients, using the Rolimeter (Aircast) [19]. A significant reduction of anteroposterior translation from a preoperative mean side-to-side laxity of 5.7 mm to 2.8 mm after surgery (*p* < 0.001) was measured in the investigation of Ventura et al. [21]. The authors implanted a fixed-bearing prosthesis in a cohort of 12 patients. All the outcome measures statistically improved at the last 7 years’ follow-up (*p* < 0.001). In a more recent study, the authors evaluated the outcomes of a cohort of 12 subjects at a 45.7-month average follow-up [22]. At that time, the OKS score reached 45.2 and the mean overall KOOS score was 86.3 (*p* < 0.001). The postoperative subgroup of KOOS regarding sport and quality of life was not statistically significant (*p* > 0.001). Leg alignment changed from a preoperative varus deformity of 3.6° ± 1° to a valgus deformity of 2.6° ± 1° [22]. In the paper of Iriberri et al., clinical scores improved significantly at the 14.5 years follow-up [20]. In particular, WOMAC, KSS, and VAS increased from the preoperative value, respectively, of 94, 59, and 8 to a postoperative value of 154, 26, and 3 (*p* < 0.01). The authors also evaluated the personal satisfaction of the patients that reached 8.8 points at the final follow-up. At the radiological evaluation, the anterior tibial translation (ATT) was measured as an indirect sign of ACL deficiency. There was not a significant reduction of ATT after surgery with a mean ATT value change from 1.6 mm to −0.9 mm (*p* > 0.37). [20] Tecame et al. [23] carried out a retrospective comparative study with 24 patients divided into two groups based on the type of implant. At the final follow-up, WOMAC index and functional and objective KSS improved significantly (*p* < 0.05), without any statistically significant differences between the two groups. In this paper, the varus deformity decreased from 4.1° ± 1.05° to 2.5° ± 1.8° and from 4.4° ± 1.3° to 2.7° ± 0.9° for Group 1 and 2, respectively. Moreover, the postoperative posterior tibial slope significantly decreased in both groups. None of these data differed statistically between the two groups. Kennedy et al. [17] studied patients that underwent staged and simultaneous ACLR and UKA. According to the purpose of this review, only data concerning the 58 subjects (76%) treated with simultaneous surgery, the largest cohort included in this review, were analyzed. At a mean 5.5 years follow-up, OKS and TAS denoted an improvement of function with a final score of 45 and 3.6, respectively. All data are summarized in Table 2.

### 3.7. Complications

According to the reported data, complications were registered in eight patients (4.7%). Among these, seven cases required surgery. One patient with lateral knee pain underwent arthroscopic external meniscus tear repair [20]. In the remaining patients, revision surgery was carried out with an overall revision rate of 3.5%. In the cohort of Tian et al. [18], there were two cases of mobile-bearing dislocations that required surgical revision and implantation of a thicker component. Revision surgery with a TKA was performed in one patient of the cohort of Ventura et al. [21] after 3 years because of progression of lateral compartment OA. A similar case was registered by Kennedy et al. [17] around 10 years after the first surgical procedure. Moreover, in the same study group, there was an infection of the implant in a diabetic patient who underwent a two-staged revision to TKA. Iriberri et al. [20] had two cases of OA progression in the lateral compartment. One patient underwent revision to TKA after 9.8 years, while the second patient was asymptomatic.

Radiolucency of the tibial component was reported in two patients. Radiolucent lines were found around the tibial component of 44% of patients in the cohort of Tinius et al. [19]. In particular, lines of 0.5 mm were seen in nine patients, of 1 mm in three patients, and of 1.5 mm in one patient. Similar images were observed near the femoral component of four patients. Tecame et al. [23] presented similar data with few cases of radiolucency line in both groups without a significant difference. They did not report early or late complications, similarly to Aslan et al. [22].

### 3.8. Methodological Evaluation

**Figure 2:**
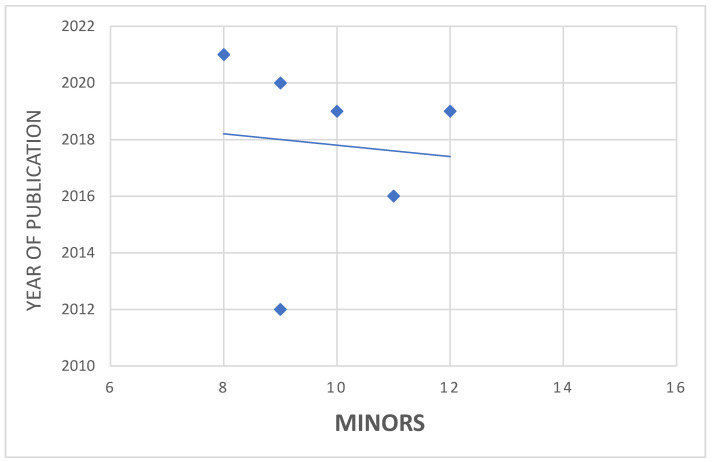
MINORS association with time of publication.

The MINORS score was used to assess the quality of the design of the studies included in the present systematic review. The average value for non-comparative studies was 10.2, ranging from 8 [22] to 12 [17]. Only the comparative investigation reached a score of 14/24 [23]. Among the papers included, only two investigations were at low risk of bias. Therefore, the overall level of methodological quality of the included studies was low. Association analysis of the MINORS score and year of publication, through the Pearson’s coefficient, showed no significant association (*p* = −0.089) (Figure 2).

## 4. Discussion

Medial knee OA is a common condition that affects from 33% to 70% of young active subjects with an untreated primary ACL injury [2]. The best option for the treatment of unicondylar bone-on-bone OA and ACL injury is still debated.

Current evidence shows that UKA in patients with stable knee has a survival rate of 96.2% at 10 years, superior to HTO [24]. In contrast, UKA has a higher failure rate in subjects with ACL insufficiency, due to eccentric loading caused by posterior femoral subluxation that can lead to implant loosening [9,12].

While different strategies have been proposed through the years to treat medial OA in ACL-deficient knees, recent studies identified UKA with ACLR as the surgical procedure with the lowest complication rate, compared to HTO combined with ACLR or TKR [7]. Moreover, UKA has the advantages of bone stock preservation, faster recovery and better long-term functional results [8].

According to the results of this systematic review, simultaneous ACLR and UKA is a safe procedure with a significant postoperative improvement of functional and clinical outcomes for patients with ACL injury that complain of knee instability and isolated medial compartment pain. This procedure is performed more frequently in young subjects with high functional requests [7]. However, age is not a clear contraindication to combined surgery. All clinical scores improved significantly at the final follow-up, with no differences between fixed-bearing and mobile-bearing implants. In particular, the mean KSS and the mean OKS improved from 106 to 163 and 29 to 44, respectively. However, the postoperative sports activity score did not significantly improve. Moreover, there was not a statistically significant difference in outcome measures between males and females and between patients with or without radiolucent lines at the final radiological evaluation. Some authors find a positive correlation between a higher posterior slope of the tibial component and better ROM.

All authors agree that patients with primary medial OA and secondary ACL deficiency are also not good candidates for this procedure. Generally, degeneration of the remaining ligament structures and advanced compartmental OA make a total knee replacement more suitable [7]. Long-term implant survival ranged from 96.5% to 100%. The overall revision rate was 3.5%. Kennedy et al. [17] studied the largest cohort (58 patients) and, at a follow-up of approximately 9 years, had a revision rate of about 3%. The main cause of revision, in general, was the progression of arthrosis in the lateral compartment which required a conversion in TKA. Tian et al. [18] reported a 7% rate of mobile bearing dislocations treated with a thicker mobile bearing during revision surgery. This data confirms the importance of proper tensioning of ACL and collateral ligaments for successful outcomes with mobile-bearing UKA. No pathological radiolucency or loosening was reported. Few authors observed physiological radiolucency at the final radiological follow-up.

In this study, unlike previous papers in the literature, only patients undergoing simultaneous ACLR and UKA were included. Indeed, some authors studied heterogeneous groups of patients underwent staged and simultaneous surgery, providing partial data that do not allow an accurate assessment. Weston-Simmons et al. [25], performing a subgroup analysis according to age (<50 vs. >50 years) and type of procedure (one-stage vs. two-stage), affirmed that there was not a significant postoperative difference in clinical and functional outcomes or implant survival rate (94.4% vs. 91.3%) between these groups. Pandit et al. [26] performed simultaneous ACLR and Oxford UKA in patients with knee pain. When instability was the main complaint, they carried out ACLR and, when ligamentous reconstruction was insufficient to improve symptoms, proceeded to subsequent UKA. In their analysis, comparing results obtained in this group (ACLR group) with a matched group of patients with intact ACL underwent UKA (ACLI group), they found that results in the ACLR group were significantly better than those achieved by the ACLI group. However, neither paper reported a summary of the subgroups’ demographic data and functional results by type of procedure, making them unsuitable for inclusion in this review.

In the literature, this is the first review focused on results of simultaneous ACLR and UKA. In previously published works, a common bias was that authors included papers in which pre- and postoperative outcomes and complication rates of simultaneous and staged procedures were not distinguishable. The simultaneous procedure is specifically indicated for patients with ligament instability and medial unicompartmental OA. Subjects with medial knee OA and secondary ACL deficiency usually have an anteromedial tibial erosion with degeneration of other articular compartments and ligamentous structures that make combined ACLR and UKA a less appropriate option. For this reason, functional outcomes of these different groups of patients cannot be compared.

The small number of low-evidence studies with a prevalent retrospective design is a relevant limitation of the present review. All papers are limited by small sample sizes that also influenced the comparative analyses when reported. Moreover, the FU time was relatively short in the majority of the papers. Furthermore, the heterogeneity of the outcome measures, the differences in follow-up periods, and the design of the prosthesis did not allow grouping the results nor a quantitative analysis. Only one comparative study was available, evaluating mobile-bearing and fixed-bearing design. There are no papers in which combined ACLR and UKA are compared to other surgical options, such as HTO or TKA. Simultaneous ACLR and UKA find application in highly selected patients and are generally performed in high-volume centers by experienced surgeons. Further studies on large cohorts with long-term follow-up are needed to confirm the efficacy and safety of this procedure, to evaluate the implant survival rate, and to compare them with other available surgical options.

## 5. Conclusions

Simultaneous ACLR and UKA is a safe procedure with an effective postoperative improvement of functional and clinical outcomes for patients with ACL injury that complain of knee instability and isolated medial compartment pain. Proper surgical indication is crucial for the success of the procedure. Further long-term studies are needed to evaluate the efficacy of this technique and to compare it with other available surgical options.

## Figures and Tables

**Table 1:** Study characteristics, demographic details and Methodological Index for Non-randomized (MINORS) score.

Authors	Year	Type of Study	LOE	Participants	M/F	Study Timelapse	Mean Age (Years ± SD)	Mean FollowUp (Years)	MINORS Score
Tinius et al. [19]	2012	RCS	IV	27	16/11	2003–2009	44 ± 3.7 (range 38–53)	4.2 ± 1.0 (range 0.75–5.9)	9/16
Tian et al. [18]	2016	PCS	IV	28	18/10	2008–2014	50.5 ± 3.5 (range 41–61)	4.3 ± 0.7 (range 1–8)	11/16
Iriberri et al. [20]	2019	RCS	IV	8	5/3	1994–2004	52 (range 42–60)	14.6 (range 9.8 –21.5)	10/16
Tecame et al. [23]	2019	RCS	III	24 Group 1: 9 Group 2: 15	20/4	2007–2013	Group 1: 47.8 (range 41–53) Group 2: 48.4 (range 43–54)	Group 1: 4.4 ± 0.7 Group 2: 3.5 ± 0.6	14/24
Kennedy et al. [17]	2019	PCS	IV	58	44/14	2001–2016	53.8 (range 41–71)	5.5 (range 1–12)	12/16
Ventura et al. [21]	2020	RCS	IV	12	8/4	2006–2010	54 ± 3.9	7.8 (range: 6–10)	9/16
Aslan et al. [22]	2021	RCS	IV	12	NA	2011–2014	NA	3.8 ± 0.4 (range 3.3–4.3)	8/16

NA: not available; PCS: prospective cohort study; RCS: retrospective case series; Group 1: mobile-bearing UKA; Group 2: fixed-bearing UKA; LOE: level of evidence; SD: standard deviation; Group 1: mobile-bearing UKA; Group 2: fixed-bearing UKA.

**Table 2:** Functional outcomes and surgical technique features.

Authors	Patients	OA Classification (n. of Patients)	Bearing and Fixation	Tendon Graft	Outcome	Preoperative (Mean)	Postoperative (Mean)
Tinius et al. [19]	27	Ahlbäck Type 4 (27)	Fixed-Bearing Cemented (27)	Four-Stranded Hamstring	KSS-KS	38.4 ± 10	83.2 ± 6.8
					KSS-FS	38.7 ± 8.8	82.7 ± 8.2
					KSS	77 ± 11.6	166 ± 12.1
Tian et al. [18]	28	NA	Mobile-Bearing Cemented (12)	Four-Stranded Hamstring	OKS	31 ± 7.1	43 ± 4.2
					KSS-KS	60.4 ± 7.1	84.5 ± 6.3
					KSS-FS	63.7 ± 6.5	86.9 ± 5.3
					TAS	4.4 ± 1.2	5.3 ± 0.8
Iriberri et al. [20]	8	Ahlbäck Type 2 (6) and Type 3 (2)	Fixed-Bearing Cemented (8)	Four-Stranded Hamstring	KSS	94 (62–165)	154 (102–200)
					WOMAC	59 (3–81)	26 (1–52)
Tecame et al. [23]	24 Group 1: 9 Group 2: 15	Kellgler–Lawrence grade 4	Mobile-Bearing (9) Fixed-Bearing (15) Cemented (24)	Four-Stranded Hamstring	KSS-KS	G1: 37.3 ± 4.3 G2: 38.6 ± 3.8	G1: 73.4 ± 9.3 G2: 77.3 ± 10.5
					KSS-FS	G1: 71.2 ± 7.4 G2: 70.2 ± 6.4	G1: 86.2 ± 6.2 G2: 84.7 ± 5.9
					WOMAC	G1: 55.78 ± 7.6 G2: 59 ± 8.1	G1: 79.3 ± 7.3 G2: 81.3 ± 7.6
Kennedy et al. [17]	58	NA	Mobile-Bearing Cementless (NA), cemented (NA)	Hamstring	OKS	29.1 ± 8	45 (41 to 47)
					TAS	2.9 ± 1	3.6 ± 2
Ventura et al. [21]	12	Ahlbäck Type 2–4 (NA)	Fixed-Bearing Cemented	Four-Stranded Hamstring	OKS	28.8 ± 10.1	42.4 ± 8.9
					KSS-KS	45 ± 12.9	75 ± 13.5
					KSS-FS	80 ± 14.2	88 ± 16.2
					KOOS	62.4 ± 8.1	80.2 ± 11.7
					WOMAC	71.9 ± 11.5	84.9 ± 9.3
Aslan et al. [22]	12	Ahlbäck Type 4 (12)	Mobile-Bearing Cementless (5), hybrid (3), cemented (4)	Four-Stranded Hamstring	OKS	29 ± 6.1	45.2 ± 3.7
					KOOS	68.5 ± na	86.3 ± na

KSS-KS: Knee Society Score-Knee Score; KSS-FS: Knee Society Score-Functional Score; KSS: Knee Society Score; OKS: Oxford Knee Score; TAS: Tegner activity scale; WOMAC: Western Ontario and McMaster Score; KOOS: Knee Osteoarthritis Outcomes Score.

**Table 3:** Rehabilitation protocols.

Authors	Rehabilitation Protocols
Tinius et al.	NA
Tian et al.	Since 6 h after surgery: partial weight bearing with assistance of walker or crutches + exercises for quadriceps + straight leg rising At the 2nd week: full weight bearing
Iriberri et al.	NA
Tecame et al.	Since 24 h after surgery: partial weight bearing with two crutches + brace with no limitation of ROM + isometric muscle exercises and restoration of ROM. In the 1st week: flexion < 90° From the 2nd week: flexion as tolerated From the 4th week: restoration of complete ROM + proprioception exercises
Kennedy et al. [3]	NA
Ventura et al.	Since 24 h after surgery: brace-free partial weight-bearing deambulation with crutches + joint motion exercises From the 4th week: full weight bearing + proprioception exercises including assisted single-leg balance and heel-to-toe walking
Aslan et al.	NA
ROM: range of motion	
